# Acute toxicity appraisal of *Saussurea heteromalla* extract and development of its chemotherapeutic herbal dosage form

**DOI:** 10.1371/journal.pone.0302657

**Published:** 2024-05-24

**Authors:** Azra Batool, Muhammad Muddassir, Basmah H. Alshammari, Tariq G. Alsahli, Gideon F. B. Solre, Yanting Liu, Muhammad Tariq Khan, Nadia Shamshad Malik, Hina Ahsan, Muhammad Tahir Khan, Muhammad Nasir Hayat Malik, Ghulam Abbas Miana

**Affiliations:** 1 Department of Oncology, The First Affiliated Hospital of Xinxiang Medical University Weihui, Henan Province, P.R. China; 2 Department of Pharmaceutical Chemistry, Hamdard Institute of Pharmaceutical Sciences, Hamdard University, Islamabad, Pakistan; 3 Department of Quality Control, Amson vaccines and Pharma, Islamabad, Pakistan; 4 Faculty of Science, Chemistry Department, University of Hail, Hail, Saudi Arabia; 5 Department of Pharmacology, College of Pharmacy, Jouf University, Sakaka, Aljouf, Saudi Arabia; 6 Tianjin Key Laboratory for Modern Drug Delivery and High-Efficiency, School of Pharmaceutical Science and Technology, Tianjin University, Tianjin, P.R. China; 7 Faculty of Pharmacy, Capital University of Science and Technology, Islamabad, Pakistan; 8 Department of Pharmaceutics, Riphah Institute of Pharmaceutical Sciences, Riphah International University, Islamabad, Pakistan; 9 Zhongjing Research and Industrialization Institute of Chinese Medicine, Zhongguancun Scientific Park, Meixi, Nanyang, Henan, P.R. China; 10 Institute of Molecular Biology and Biotechnology (IMBB), The University of Lahore, Lahore, Pakistan; 11 Faculty of Pharmacy, The University of Lahore, Lahore, Pakistan; University of Agriculture Faisalabad, PAKISTAN

## Abstract

Ethnopharmacological relevance of *Saussurea* species for anti-cancer compounds instigated us to develop chemotherapeutic herbal tablets. This study was an ongoing part of our previous research based on the scientific evaluation of *Saussurea heteromalla* (*S*. *heteromalla*) for anti-cancer lead compounds. In the current study, *S*. *heteromalla* herbal tablets (500 /800 mg) were designed and evaluated for anti-cancer activity. Arctigenin was found as a bioactive lead molecule with anti-cancer potential for cervical cancer. The *in vitro* results on the HeLa cell line supported the ethnopharmacological relevance and traditional utilization of *S*. *heteromalla* and provided the scientific basis for the management of cervical cancer as proclaimed by traditional practitioners in China. LD50 of the crude extract was established trough oral acute toxicity profiling in mice, wherein the minimum lethal dose was noticed as higher than 1000 mg/kg body weight orally. Chromatographic fingerprint analysis ensured the identity and consistency of *S*. *heteromalla* in herbal tablets in terms of standardization of the herbal drug. About 99.15% of the drug (*S*. *heteromalla* crude extract) was recovered in herbal tablets (RSD: 0.45%). *In vitro* drug release profile was found to be more than 87% within 1 h, which was also correlated with different mathematical kinetic models of drug release (r^2^ = 0.992), indicating that drug release from matrix tablets into the blood is constant throughout the delivery. The dosage form was found stable after an accelerated stability parameters study which may be used for anti-cervical cancer therapy in the future, if it qualifies successful preclinical investigation parameters.

## Introduction

Medicinal herbs and their derived phytocompounds are finding upsurge relevance today as helpful complementary curing entities for different medical-related conditions, specifically diabetes, and cancer. An increased global consumption of herbal medicines (HM) can be experimental among cancer patients and the surviving people to improve their health and quality of life and minimize conventional therapy-provoked toxicities [1,2]. Several clinical studies have proven the usefulness of HM alone or as an adjuvant with conventional therapeutics to regulate cancer patients’ immune systems and survival [3]. The secondary metabolites found in plant crude extracts, such as flavonoids, glycosides, alkaloids, terpenoids, etc., are diverse and have several benefits since they are soluble and pure, which significantly reduces the dosage concentration needed. Aqueous or alcoholic crude extracts can be regarded as HMs as they contain active constituents. They are incorporated to create a variety of dosage forms, such as alcoholic tinctures, lotions, tablets, capsules, ointments, emulsions, and decoctions, as well as herbal extracts and powders [2].

*Saussurea heteromalla* (Synonyms; *Himalaiella heteromalla*, *Saussurea candicans*, *Jurinea heteromalla*) belongs to the family *Compositae*, the only specie distributed in abundance among the regions of Himalaya, tropical and subtropical. It is a rich source of medicinally active products, including sesquiterpene lactones (chlorojanerin), terpenoids, steroids, and lignans (arctiin, arctigenin). Therefore, the plant is accredited to impart its distinct role in phytochemistry and ethnopharmacology as an anti-cancer folk remedy. It is also abundantly bred in the Islamabad region of Pakistan and some regions of neighbouring China; therefore, based upon scientific principles, *S*. *heteromalla* indigenous to China and Pakistan was assumed to be a rich source of therapeutically active compounds. In our previous study, we evaluated *S*. *heteromalla* crude extract for antioxidant, cytotoxic, antifungal, anti-diabetic, and antibacterial potentials [4]. We isolated, characterized, and identified three compounds arctigenin, arctiin, and matairesinol and amongst them, arctigenin was documented as an active molecular lead with significant anti-cancer potential [4,5]. In current study, we developed a chemotherapeutic herbal dosage (Tablets) from *S*. *heteromalla* crude extract after evaluating its anti-cancer potential against HeLa cell line and normal murine fibroblast (NIH) cells and also investigated the qualitative and quantitative stability studies.

## Materials and methods

### Collection of plant

*S*. *heteromalla* whole plant was collected from Islamabad (Pakistan), identified by Dr. Ghulam Abbas Miana, and authenticated by Dr. Muhammad Zafar. The dried plant specimen was deposited in the herbarium of Quaid-e-Azam University Islamabad, Islamabad, Pakistan (Reference No: ISL-130608). Following the standard procedure of plant extract, the whole plant’s greenish crude methanolic extract (CME) was isolated using percolation. *S*. *heteromalla* CME was dried and concentrated through a rotary evaporator under reduced pressure. The % yield of crude CME was found to be 63% which was calculated through the following formula;

%Extractrecovery(%w/w)=Driedextractweight/Powderedplantmaterialweight

### Preliminary phytochemical screening

Phytochemical analysis CME was performed to observe the presence of flavonoids, alkaloids, terpenoids, carbohydrates, proteins, saponins, tannins, phenolic compounds sterols, lactones, triterpenes, sesquiterpenes, and glycosides using standard methods [6].

### Biological evaluation

#### Cytotoxicity evaluation (in vitro anti-cancer activity)

The *in vitro* cell toxicity profiling of *S*. *heteromalla* extract was conducted by performing MTT (3- (4, 5-dimethyl thiazol-2-yl)-2, 5-diphenyl tetrazolium bromide) assay using HeLa and NIH cell lines [7] as previously described [4]. IC_50_ values (extract concentration showing 50% of cells viability) were recorded by sigma plot.

Briefly, NIH and HeLa cells were cultured in a growth medium (phosphate buffer (10%) and Dulbecco’s modified eagle medium) at 37°C. NIH and HeLa cells’ density was attained up to 6x10^3^ cells /well in 96 wells microplate in 200 μl growth medium. The extract of *S*. *heteromalla* CME was dissolved in DMSO to form a stock solution (10 mM) that was successively diluted with culture medium to constitute various concentrations i.e., 25, 50, 100, and 200 μg/ml. Each concentration was supplemented to the cultured cells after 24h. The growth medium was removed after 72h. MTT solution (0.5mg /ml, pH = 7.4) was added to each well of the microplate and left for two h for additional incubation to form a formazan substance (purple color) in the viable cells. Formazan crystals were removed from the microplate and treated with lysis solution (0.1% Nonidet P40 in ethanol + 4mM HCl). The absorbance of the color solution was measured through a microplate reader (EL x 808, BioTek, USA) at 570 nm. Negative control was achieved by incubating cells with medium and dimethyl sulfoxide (DMSO) only. Triplicate Analysis was performed for all sample concentrations, and data was articulated as the mean ± SEM. The *in vitro* cell toxicity behavior of arctigenin was investigated by performing semi-automated testing method Sulforhodamine-B (SRB) assay using HeLa cell line as described by Batool et al,. 2022 [5]. Cyclohexamide and doxorubicin were taken as reference standards for NIH and HeLa cell lines, respectively. % cell inhibition and IC_50_ of the extract and standards were determined using a formula.


$$\%\;cell\;inhibition=\frac{100-(\mathrm{Test}\;\mathrm{compound}\;\mathrm{absorbance}-\mathrm{blank}\;\mathrm{absorbance})x100}{\left(\mathrm{Control}\;\mathrm{absorbance}-\mathrm{blank}\;\mathrm{absorbance}\right)}$$

#### Ethical approval

Experiments were performed according to guidelines of ARRIVE and were approved by Institutional Research Ethics Committee of Department of Pharmaceutical Chemistry, Hamdard Institute of Pharmaceutical Sciences, Hamdard University, Islamabad, Pakistan (Approval number: AAEC-08/2020).

#### In-vivo oral acute toxicity

The acute toxicity (LD_50_) test manifests the dosage range that could be administered approximately and establishes the medicinal product’s therapeutic index or safety margin. As a general rule, acute toxicity concludes LD_50_ value on experimental bases in animals. To estimate the therapeutic index of *S*. *heteromalla* extract, its *in-vivo* oral acute toxicity profiling was carried out. The LD_50_ determination was performed in female *albino* mice aged about eight weeks and weighing 30–35 g. Mice were kept in a well-ventilated animal house (Amson vaccines & Pharma, microbiology laboratory Islamabad, Islamabad Pakistan) for five days following the standard conditions of room temperature, humidity, and food, i.e., 58 ± 5%, under 12h light/dark cycle, *ad libitum* feeding and water. Five groups of animals (6 mice in each) were arranged. The control (group-I) was provided only food and water *ad libitum*. In contrast, the remaining four experimental groups additionally received *S*. *heteromalla* extract at variable doses, i.e., 500 mg/ kg (group- II), 1000 mg/kg (group- III), 2000 mg/kg (group- IV) and 3000 mg/kg (group- V). Each dose was formulated by dissolving *S*. *heteromalla* extract (500–3000 mg) in normal saline (10 ml/kg body weight) and was given orally. Each experimental and control group was continuously administered extract dose and normal saline for seven days. Animals were kept under critical observation for clear toxicity signs or other related behavioral changes, including tremors, restlessness, weight loss, diarrhea, sluggishness, and paralysis at specific intervals for the early four hours following oral administration of the crude extract. From experimental data, LD_50_ of the *S*. *heteromalla* extract was calculated by the Arithmetical Method of Karber 1931, Reed and Muench 1938 [8]. Animals which showed signs of respiratory cessation and fainting of heart beat were euthanized by intra-peritoneal injection of pentobarbital sodium (200 mg/kg). Care was taken to minimize the sufferings of animals [9].

#### Preparation of herbal tablets

*S*. *heteromalla* herbal tablets (SH-ht) were formulated after experimentally proving the anti-cancer traditional implementation through a scientific approach. The CME was dried by keeping it under vacuum desiccators to form a powder; excipients were taken by the pharmaceutical company (Amson vaccines & Pharma Islamabad, Islamabad-Pakistan). Tablet composition was adjusted for the batch size of 125 tablets (500 mg CME powder + 300 mg excipients = 800 mg/tablet, theoretical weight). Granules of the powdered material were prepared by mixing and slugging the required excipients (stearic acid, colloidal silicon dioxide, microcrystalline cellulose (Avicel 102), polyvinyl polypyrollidone and talcum) and CME powder for the required batch size. Excipients were added according to their specified percentage in practice by pharmaceutical companies. Granules were compressed into tablets (800 mg) by a rotary “single punch machine”.

### Preformulation studies of Granules/ powder blend

#### Chromatographic fingerprint analysis

HM contains multiple classes of phytochemicals; each one contains several constituents that might be relevant to the medicine’s putative action. Therefore, analytical techniques that deal with a collection of compounds, e.g., their detection, particular percentage, and ratios, offer an additional rational approach to assessing the authentication and quality. The herbal drug, either single or in combination, consists of a myriad of components in which no single active ingredient is responsible for exerting overall efficacy. This built up a challenging situation to establish standards for quality control of raw materials and finished form herbal drugs standardization as well. WHO has accredited this problem a draft “Strategic *Plan for Regional Traditional Medicine”* [10]. Now, commonly in a practical way among herbal products, there is a selection of one or more components as either active base or “markers” for identification and quality evaluation. Chromatographic fingerprint analysis was applied for the determination of the identity, consistency, and stability of HM by performing “Standard solution (crude extract) and sample (SH-ht) assays [11].

#### Qualitative testing

**Fig 1:**
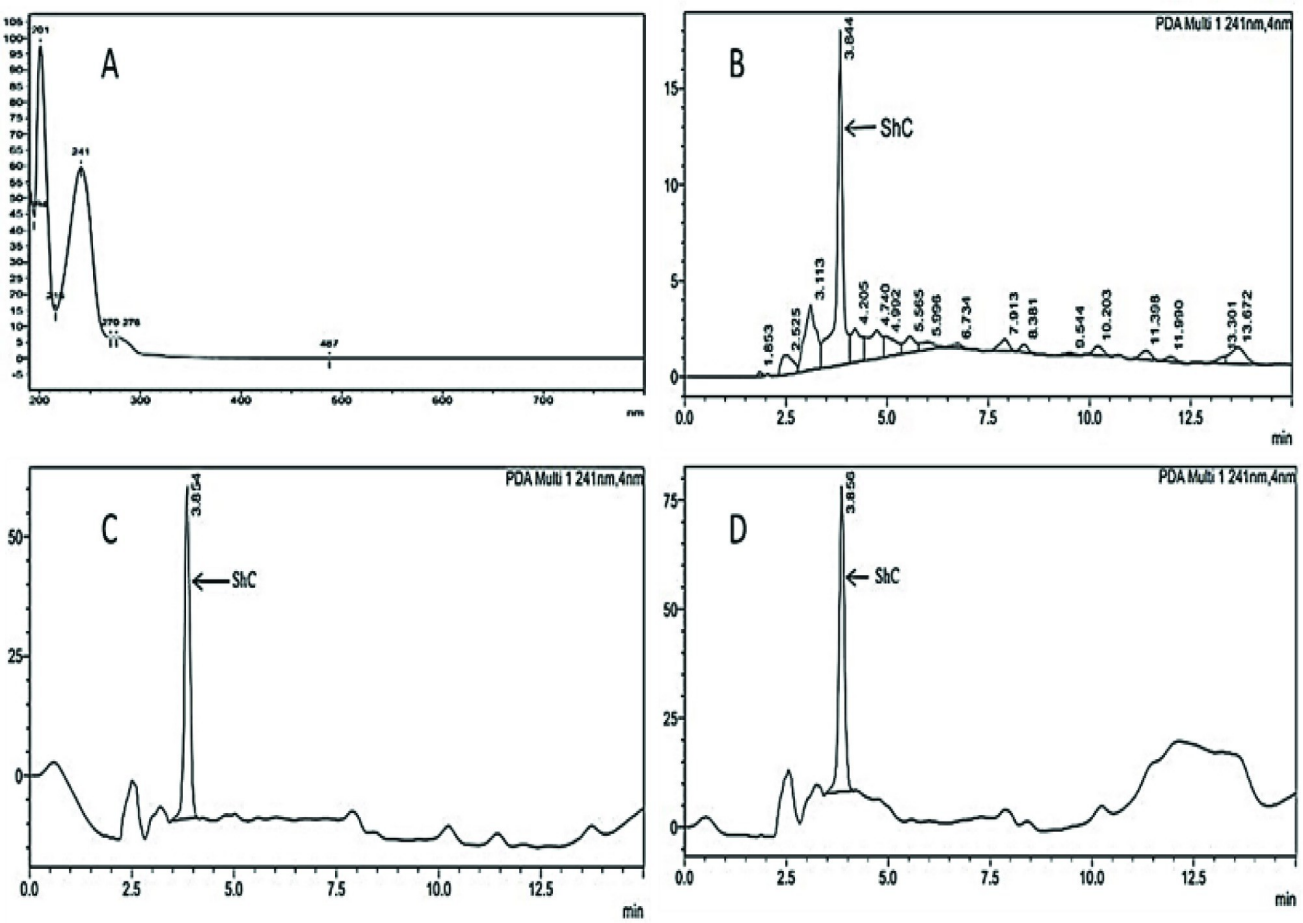
UV-spectrum of *S*. *heteromalla* crude extract at wavelengths 201 and 241 nm in 190–800 nm UV range **(A)**. HPLC chromatogram of *S*. *heteromalla* crude extract showing multiple detected compounds at different retention times **(B)**. *S*. *heteromalla* crude extract (standard) chromatogram, (**ShC**) was selected as standard marker or base peak **(C)**. *S*. *heteromalla* Crude extract sample (tablets) chromatogram **(D)**.

Fingerprint chromatographic procedure was utilized for *S*. *heteromalla* crude extract using a quaternary gradient HPLC system (Shimadzu 20A Prominence series, Japan) with a photo-diode array (PDA) detector. Reversed-phase silica gel columns of different lengths and diameters were utilized. The optimum resolution was obtained with C-8 (octasilyl) 250 mm × 4.6 mm column length, 5 μm particle size, and mobile phase composition of acetonitrile: methanol: water (50:30:20). Analysis was performed at ambient temperature and 1.0 ml/min flow rate. The solution of crude extract was scanned in the UV range of 190–800 nm and showed maximum resolution at 201 and 241 nm wavelengths (Fig 1A). About 50 μl of SH crude extract (0.02% in methanol) was injected in triplicate, and the relative standard deviation (%) was calculated. A fingerprint chromatogram of crude extract with multiple peaks at variable retention time was obtained. The highest peak **(ShC)** at a retention time of 3.84 min was selected as the “marker peak” for assay and dissolution quantification of herbal tablet (SH-ht) dosage form.

#### Angle of repose

It was checked through the fixed funnel method. The 10 g of powder blend was poured into a glass funnel to form a heap of the blend. The lower tip of the glass funnel was 2 cm high (h) from the ground. Diameter of the pile (heap circle) was measured from there different points, and the average diameter (d) was determined, similarly average radius (r) of the heap blend was determined from the diameter (diameter /2 = radius). Following formula was used to calculate angle of repose:

Tanθ=Heightofthepile(h)/Radiusofthepile(r)

#### Bulk density

A 5g powder blend (Mass) was weighed accurately and gently filled into a 20 ml measuring cylinder with no compacting. The apparent unsettled volume (V_0_) of the graduated cylinder was measured to calculate the bulk density (g/ml) using the formula:

$$Bulk\;Density=Mass/V_{0}$$

#### Tapped density

According to the USP method, tapped density was measured by gently pouring 5g powder sample into a 20ml measuring cylinder without tapping. Then the cylinder was tapped mechanically by tapped density tester that allowed suitable fixed dropping distance and drop/tapping rate of the powder [12]. The tapped volume of the powder blend was noted to calculate tapped density as follows:

Tapped Density = M/V_f_

M = mass (g)

V_f_ = final compact volume (cm^3^)

#### The compressibility index

The compressibility index (CI), also known as Carr’s index, is the tendency of a powder to consolidate [13]. Data obtained from testing bulk density and tapped density was used to measure the compressibility index by following Equation: where V_o_ = untapped apparent volume, V_f_ = tapped apparent volume.

(CI) % = 100 ×V_o_- V_f_ / V_o_

#### Hausner’s ratio (HR)

The Hausner ratio has a close relation to CI; it was calculated as follows:

Hausner ratio = V_o_/V_f_

V_0_ = apparent unsettled volume (cm^3^)

V_f_ = tapped final volume (cm^3^)

#### Loss on drying

Loss on drying (LOD) is a physical test to determine the moisture in the tablet’s formulation. It is expressed as a percentage w/w resulting from the evaporation of water and volatile matter from granules. LOD of well-mixed granules was determined at 105°C using a Sartorius Moisture Analyzer utilizing 1.0 g of weight.

### Characteristics of tablets

#### Identification

In the HPLC chromatogram, the retention time of the main peak (**ShC**) of the tablet sample solution was concordant with that of the crude extract standard solution obtained during assay performance.

#### Weight variation

The physical appearance was brownish green colored oblong tablets. The weight of twenty tablets was measured between 798–802 mg. All tablets passed a weight variation test according to the USP limit specification ± 5%.

#### Friability

“Pharma test Germany” friability tester was used to record the tablets’ friability. Twenty tablets were weighed and loaded in the friabilator that was rotated for 4 min (100 rotations, 25 rpm); the tablets were removed, de-dusted, and weighed again to calculate the percentage of friability [14].

Friability percentage = Initial weight − final weight / Initial weight × 100

#### Hardness

The tablet must be strong and resistant to withstand mechanical shock during manufacturing, packaging, and shipping handling. Thus, hardness is the strength of crushing [14]. Ten tablets’ hardness was measured using a Pharma Test digital hardness tester in kg/cm^2^.

#### Disintegration time (DT)

The important prime factor toward the dissolution of the tablet is its breakdown and conversion to smaller particles through the process of disintegration. Using disintegration apparatus (Pharma test, Germany) DT of the six tablets was determined in water at 37 ± 1°C [14].

#### Thickness

Tablet thickness was measured with PharmaTest automated tablet diameter, thickness, and hardness tester (Model: PTB 311).

#### Dissolution testing

*In vitro*, using the paddle method apparatus rotating at 50 rpm at 37°C ± 0.5°C for one hour, the drug release percentage was monitored at 10, 20, 30, 45, and 60 min. This was found to be not less than 75% of the labeled amount dissolved in one hour, which qualified the standard USP limit.

### Quantitative testing

#### Assay of crude extract tablet / finished dosage form

The quantity of crude extract in tablets was determined through HPLC. The assay of tablets was performed on a low-pressure quaternary gradient liquid chromatographic system (Shimadzu 20A series SIL 20A, Japan) using an octasilyl silica gel reversed-phase column (250 mm × 4.6 mm, 5 μm, Phenomenex-Luna,) attached to photo-diode array (PDA) detector. The mobile phase (consisting of acetonitrile: methanol: water, in the ratio of 50:30:20) was pumped at a flow rate of 1.0 ml/min, and a detection wavelength of 241 ± 4 nm was programmed at ambient temperature (25 ± 1°C). Solution(s) of crude extract and powdered tablets (0.2 mg/ml) were prepared in methanol and filtered through a 0.22 μm syringe filter. About 50μl of standard solution (in triplicate) and sample solution (in duplicate) were injected, and marker peak areas (**ShC**) of standard solution and sample solutions were calculated. High precision of percentage was achieved (RSD <2%). Assay results (Table 5) were deduced using the following formula:

%Assay=Peakareaofsamplesolution/Peakareaofstandardsolutionx100

#### Dissolution profile (In vitro drug release of tablets)

In the dissolution process, a solid enters the liquid to form a solution. The dissolution rate refers to the drug concentration released or goes into solution per unit of time under standardized conditions, i.e., liquid-solid interface, solvent composition and temperature. *In vitro* dissolution is a highly significant test and quality control tool for predicting the active substance’s bioavailability. However, the most direct assessment of the drug release from tablet formulation into the blood is accomplished via *in vivo* bioavailability parameter measurement [15].

#### Dissolution procedure

**Fig 2:**
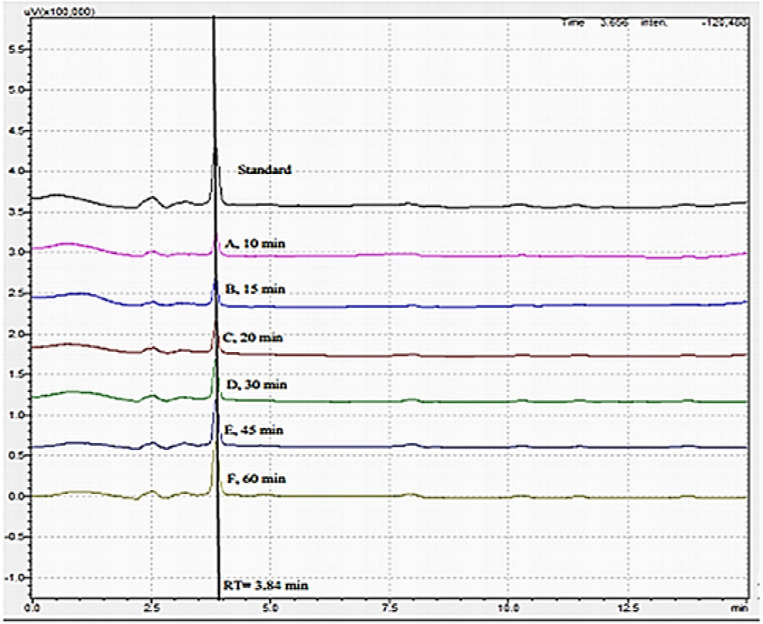
Stacked chromatograms of standard and sample dissolution at different time intervals.

The dissolution profile of six herbal tablets (Table 6) was performed on Pharmatest PTWS 1220, Germany. Phosphate buffer pH 6.8 (dissolved 6.805 g monobasic potassium phosphate and 0.9 g of Sodium hydroxide in 1000 ml of water) was used as dissolution medium (500ml) using the paddle (Apparatus II) method, rotating at 50 rpm at 37°C ± 0.5°C. The sample was taken at different time intervals (10, 15, 20, 30, 45, and 60 min) by withdrawing 10 ml from each vessel. 10 ml of medium (already maintained at 37°C) was added to vessels at each time interval to replace the withdrawn sample. A 50 μl of filtered (0.45 μm syringe filter) standard solution (0.2 mg/ml) and sample solutions were injected and “marker” peak areas (**ShC**) were calculated at different time points using chromatographic conditions as described in assay of crude extract tablets (Figs 1D and 2). Acceptance criteria were not less than 75% of the claimed amount of crude extract from herbal formulation should dissolve in 60 minutes [15].

#### Kinetic release studies

Data collected from *in vitro* herbal drug release profile was incorporated into different kinetic study models, including Zero order, first order, Higuchi and Hixson Crowell models to evaluate the kinetic release of drug in terms of r^2^ Table 7 [16].

#### Zero order model

**Fig 3:**
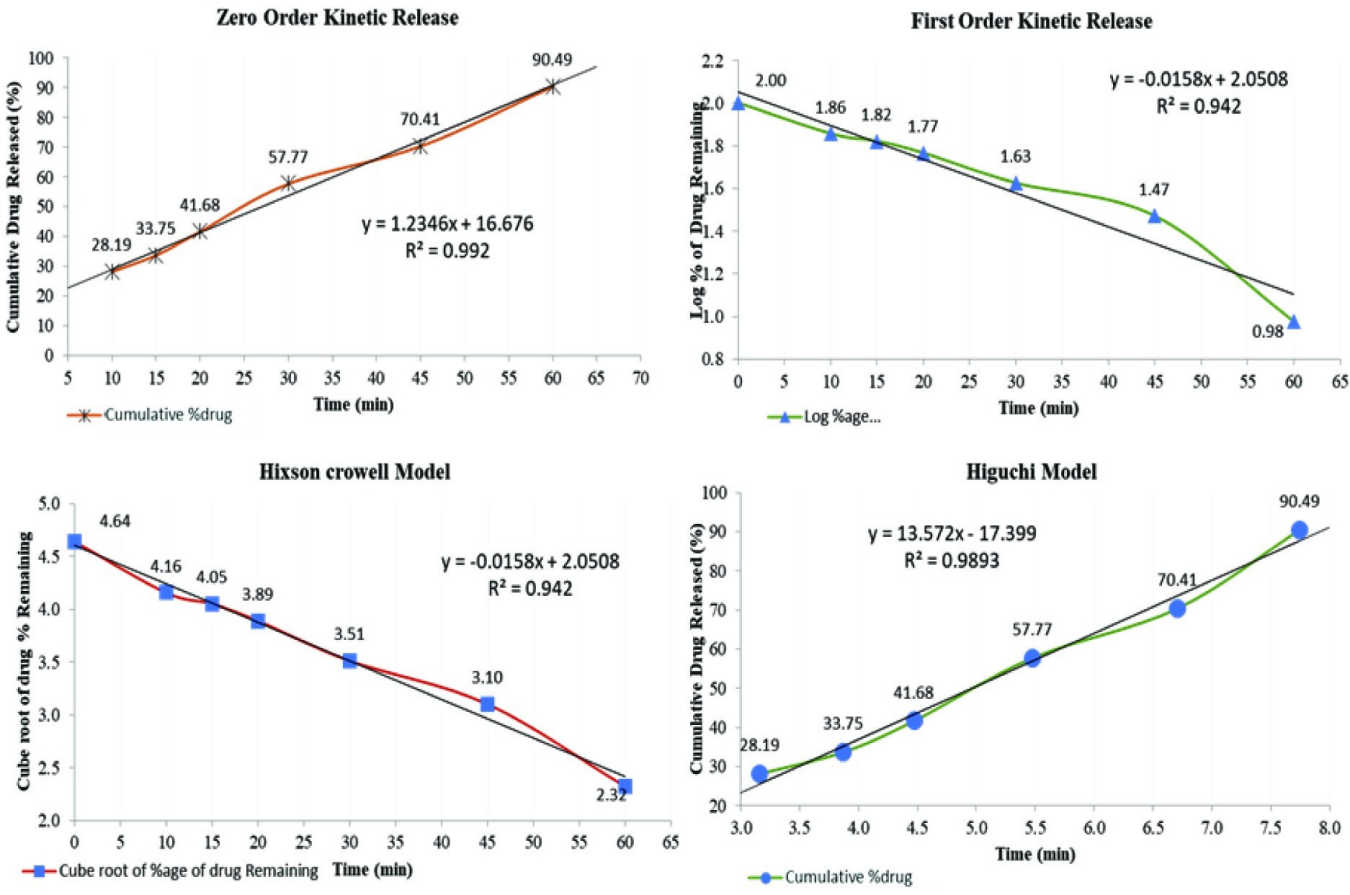
Four different kinetic models of release of *Saussurea heteromalla* extract tablets.

Data retrieved from in-vitro dissolution analysis is plotted versus time, i.e., cumulative drug release against time, as given in Fig 3 [17].

According to the pharmacokinetics principles, drug release from any dosage form can be represented by following equation:

C_0_-C_t_ = K_0_t

C_t_ = C_0_+K_0_t

C_t_ = drug quantity released at time t, C_0_ = initial drug concentration at time t that is zero, K_0_ = zero-order rate constant. Hence, zero order kinetics demonstrates the procedure of regular drug release from a dosage form, and the drug concentration in the blood is maintained constant throughout the drug distribution.

#### First order model

It creates the relationship between percentages of drug release versus time [17]. Following equation displays the integration and reorganization of the data.

Log C_t_ = Log C_0_ + K_t_/2.303

Where; K = first order rate equation, C_0_ = drug initial concentration, C_t_ = drug remaining at any specific time interval, and t = time in hours.

#### Higuchi model

It is the most outstanding pharmacokinetic model, which intricate drug dissolution and diffusion dependent on drug concentration [18]. Following equation is the simplified form of the Higuchi model.

Q = K_H_ × t^1/2^

Where; Q = Cumulative quantity of drug released after time t, K_H_ = rate constant of Higuchi release, and t^1/2 =^ square root of time.

#### Hixson-Crowell model

This cubic root law illustrates the release from matrix systems that change in surface area and drug particle diameter [19]. Hixson-Crowell establishes a correlation between drug release viz. times represented by following equation.

W_0_^1/3^- W_t_^1/3^ = K_HC_ t

This equation interprets dissolution data collected from conventional, immediate release, or dispersible dosage forms. Therefore, if the correlation coefficient (r^2^) of the abovementioned equation 5 is higher, it can be concluded that changing surface area throughout the dissolution process has a remarkable effect on drug release.

### Accelerated stability study of herbal tablets

Depending on environmental conditions, the world has been categorized into I-IV zones for stability testing. Long-term and accelerated stability testing parameters have been established based on mean relative humidity and annual temperature data. Association of Southeast Asian Nations (ASEAN) provided the guidelines for stability testing, which ensure quality consistency and maintenance of final herbal dosage form (traditional medicines) in recommended packages in terms of storage conditions and times. In accelerated studies product is studied for six months. Because Pakistan is present in zone–IVa and (hot and humid) and China in zone II, therefore following the ASEAN guidelines, the stability studies of herbal tablets were performed to ensure product safety under accelerated conditions, i.e., 40°C ± 2°C/75 ± 5% RH (Pakistan zone -IVa, China zone II). For this purpose, the formulated *S*. *heteromalla* herbal tablets were placed at 30°C ± 2°C/ 65 ± 5% RH and 40°C ± 2°C/75 ± 5% RH for six months. The hardness, friability, content uniformity, *in-vitro* disintegration time and drug release were evaluated [20].

### Statistical evaluation

GraphPad Prism 8 was used to perform statistical analysis. IC50 were calculated using Dose-response inhibition module. T-test was used to compare the difference between two groups while One-way ANOVA was used to analyze the experimental data for 3 or more groups at confidence level of 0.05. Data were expressed as Mean ± SEM and p ≤ 0.05 was considered as significant.

## Results

### Preliminary phytochemical screening

*S*. *heteromalla* CME possessed the following phytochemicals (Table 1).

**Table 1:** Phytochemicals present in *S*. *heteromalla* CME.

Phytochemical (s)	Test (s)	Indication	Result
Alkaloids	Mayer’s reagent test	Reddish brown precipitates	+
Tannins /Phenolics	FeCl_3_ test	Blue/black Precipitates	+++
Flavonoids	NaOH test	Yellow color	+++
Glycosides	FeCl_3_ test	Rose pink	+++
Terpenoids	Salkowski test	Reddish brown ring	+++
Saponins	Froth test	Unstable froth after 10 min	−
Carbohydrates	Molisch’s test	Brick red color	+++
Steroids	H_2_SO_4_ test	Red color	+++

### Biological evaluation

#### In-vitro anti-cancer activity / cytotoxicity assay

In comparison with doxorubicin inhibition (95.90; IC_50_ value 0.2 ± 0.03 μg/ml), the *S*. *heteromalla* CME demonstrated potential inhibition (28.63 ± 1.69, 45.17 ± 0.85, 61.17 ± 2.45, 77.28 ± 1.53) after a 48 h of exposure in a concentration-dependent manner at all tested concentrations (25, 50, 100, 200 μg/ml) and at different efficacies, respectively, with IC_50_ of 62.13 μg/ml on Hela cell line. The inhibition of NIH/3T3 cells growth was fewer with more IC_50_ (68.4μg/ml) compared to cycloheximide standard (IC_50_; 25.48 μg/ml). On the other hand, arctigenin exhibited moderate cytotoxicity against HeLa cells (IC50: 11.04 μg/ml).

#### In-vivo oral acute toxicity studies and determination of dose

It was observed that groups IV and V receiving *S*. *heteromalla* CME above 1000 mg/kg body weight (b.w) displayed a remarkably significant decrease (*P* < 0.05, 26.58 ± 0.023 & 25.7 ± 0.011) in body weight as compared to a control group- I (Table 2A). LD_50_ value of the *S*. *heteromalla* CME on female mice was found to be 1500 mg/kg b.w. The herbal extract did not exhibit any symptoms of toxic effects up to 1000 mg/kg. The minimum lethal dose was higher than 1000 mg/kg when administered orally in female mice, while 3000 mg/kg was expressed as acute lethal (Table 2B). Since its lethal dose (LD_50_) is less than 5000 mg/Kg, extracting *S*. *heteromalla* is considered toxic (Loomis & Hayes, 1996). LD_50_ of *S*. *heteromalla* CME was determined as 1500 mg/kg body weight. Hence, any dose up to 1400 mg/kg can be used; therefore, 100 mg/kg was expressed and selected as ED_50_. At the same time, 1000 mg/kg was designated as ED_50_ (max). The minimum drug concentration was chosen to produce therapeutic action for commercially economical herbal formulation and further research.

**Table 2:** Changes in body weight treated with *S*. *heteromalla* CME for seven days.

**(A) Body Weight (Mean ± SEM) (T**reated with *S*. *heteromalla*)					
**Dose (mg/kg) b.w**	Group-I (Control)	Group-II (500 mg)	Group-III (1000 mg)	Group-IV (2000 mg)	Group-V (3000 mg)
**Weight (g) before drug**	31.4 ± 0.25	31.30 ± 0.25	31.7± 0.28	31.2 ± 0.25	31.8 ± 0.17
**Weight (g) after drug**	31.6 ± 0.010	31.8 ± 0.010	31.2 ± 0.010	26.58± 0.023	25.7 ± 0.011
**(B) Dose Vs mortality and LD**_**50**_ **value of *S*. *heteromalla* CME**					
**Dose (mg/kg) b.w**	Group-I Normal saline	Group-II (500 mg)	Group-III (1000 mg)	Group-IV (2000 mg)	Group-V (3000 mg)
**No of animals**	6	6	6	6	6
**Death /Mortality**	0	0	0	6	6
**LD**_**50**_ = 1500 mg/kg					
**ED**_**50**_**(max)** = 1000 mg/kg					

*p* <0.05 (S), Values are expressed as ± SEM (standard error of mean), S = significant. B.w: body weight.

### Preformulation studies/evaluation of powder blend (granules)

#### Qualitative testing

*Fingerprint chromatographic analysis*. HPLC fingerprint analysis of *S*. *heteromalla* crude extract provided a high-resolution chromatogram at wavelengths 201 and 241 nm in 190–800 nm UV range. The chromatogram of extract comprised multiple peaks corresponding to their compounds relative to their AUC at variable retention times achieved (Fig 1). Peak **(ShC)** with a maximum height at a retention time of 3.84 min was selected as a “**marker peak**” that served the purposes of identification, quality assessment, and standardization of finished herbal tablet (SH-ht) dosage form during its assay.

#### Granules characteristics

Granules (angle of repose, bulk density, tapped density, compressibility index, Hausner’s ratio, and loss on drying) and formulated herbal tablets characteristics data are mentioned in Table 3.

**Table 3:** Characteristics of powder blend, formulated herbal tablets, and flow ability scale range.

Characteristics of Powder blend	Bulk density (g/ml)	Tapped density (g/ml)	Carr’s index (%)	Hausner’s ratio	Angle of Repose (°)	
	0.46	0.52	8	1.21	32.1	
**Characteristics of formulated Tablets**	Hardness (kg/cm^2^)	Thickness (mm^2^)	Weight Variation (%)	Friability (%)	Disintegration Time (min)	Dissolution
	6.2 to 7.0	6.18 ± 0.05	1.28 ± 0.03	0.36 ± 0.14	8.4 ± 0.11	NLT 75% of the labeled amount dissolved in 60 minutes
**Flowability Scale**	Flow Parameter	Compressibility Index		Hausner Ratio	Angle of Repose	
	Excellent	< 10		1 to 1.2	< 20	
	Good	11 to 15		< 1.25	21–30	
	Fair	< 15		< 1.25	> 30	
	Pass	20–25		≥ 1.34	30–40	
	Poor	20–25		≥ 1.34	30–40	
	Very poor	> 30		< 1.45	—	
	Rejected	>38		>1.6	—	

### Evaluation of formulated tablets

#### Identification

In the HPLC chromatograms, the retention time of the main peak (**ShC**) of the tablets sample solution was concordant with that of the crude extract standard solution obtained during the assay performance of finished crude extract tablets in quantitative testing (Fig 1).

#### Dissolution time

*In vitro*, using paddle method apparatus, rotating at 50rpm, 37°C ± 0.5°C for one hour, the drug release percentage was monitored at 10, 20, 30, 45, and 60 minutes. A drug dissolved in one hour qualified the USP’s generalized tolerance limits. Not less than 75%, as mentioned in Table 3 [15].

### Quantitative testing (standardization of finished herbal dosage form)

#### Standard and sample (SH-ht) solutions assay

The percentage of *S*. *heteromalla* crude extract in tablets was determined through HPLC. The retention time for marker peak (**ShC**) in chromatograms of standard and a sample (tablets) solution was 3.84 minutes with a mean area under the Curve (AUC) 645952 and 640461, respectively. Precision was expressed as (RSD) Relative standard deviation (1.15%) obtained with triplicate injection of *S*. *heteromalla* crude extract standard solution. Percentage assay result of *S*. *heteromalla* crude extract in the herbal tablet was 99.15% with RSD 0.45%, which manifested high precision accuracy, as shown in Table 4.

**Table 4:** Composition and Assay results of *S*. *heteromalla* extract tablets.

Materials	Quantity (mg)	Quantity (g)
	Unit Tablet	125 Tablets
*S*. *heteromalla* CME Powder	500	62.5
Stearic Acid	20	2.5
Colloidal Silicone Dioxide	7	0.88
Talc	22	2.75
Microcrystalline Cellulose	225	18.13
Polyvinyl Polypyrollidone	26	3.25
Total Weight	800	100
**Assay results of *S*. *heteromalla*extract tablets**		
**Replicate**	**Area under Curve (mAU)**	
	**Standard (crude extract)**	**Sample (Tablet)**
Replicate 1	654214	637425
Replicate 2	639751	640758
Replicate 3	643892	643201
Mean	645952	640461
Standard Deviation	7448.4	2899.4
RSD (%)	1.15	0.45
**%age Result**		**99.15%**

#### Dissolution profile (in vitro drug release of tablets)

Dissolution of *S*. *heteromalla* crude extract herbal tablets was performed in phosphate buffer and sampling from each vessel was performed at specified time intervals. The AUC of each sample was measured, and the mean (AUC) of six tablets was calculated. The % drug dissolved at each time interval was deduced from the peak area of the standard solution of *S*. *heteromalla* crude extract (0.2 mg/ml). The release pattern of crude extract from herbal tablets was 28.13%, 33.45%, 41.08%, 56.06%, 69.13%, and 87.48% at 10, 15, 20, 30, 45, and 60 min, respectively. The AUC of standard/sample and % release is presented in Table 5.

**Table 5:** Drug release profile of *S*. *heteromalla* extract tablets (dissolution).

Standard		Sample								
Replicate	AUC Standard (crude extract)	Time (min)	AUC Sample (Tablets)						Mean	Release (%)
			Sample 1	Sample 2	Sample 3	Sample 4	Sample 5	Sample 6		
1	663639	10	187339	187255	185420	186540	186547	188660	186960	28.13
2	661422	15	222245	232680	217836	218964	204569	237484	222296	33.45
3	668833	20	272479	263647	272254	274452	276584	278924	273057	41.08
**Mean AUC (Standard)**		30	376374	394256	395686	364879	364215	364120	376588	56.66
		45	456177	472124	468620	452214	459864	447622	459437	69.13
664631		60	584620	610045	592642	574251	569267	557542	581395	87.48

Stacked chromatograms of different time points show increased peak height. The drug release profile of *S*. *heteromalla* crude extract tablets show a continuous release of the drug in phosphate buffer, achieving the desired amount of drug dissolved in one hour (87.48%) (Fig 2). Dissolution test results comply with the general acceptance limits of not less than 75% drug dissolved at the specified time of one hour [15].

#### Evaluation of kinetic release mechanism

Data obtained from the *in-vitro* drug release profile was correlated with different mathematical kinetic models of drug release, interpreted as graphical representation and was calculated by correlation coefficient (r^2^) as shown in Table 6.

**Table 6:** Data from different models representing r^*2*^, *slope* and intercept.

Model	r^2^	*Slope*	Intercept
Zero order model	0.992	1.234	16.67
First order model	0.942	-0.0158	2.0508
Hixson-Crowell model	0.942	-0.0158	2.0508
Higuchi model	0.989	13.57	17.39

Above comparison Table 7 showed that zero-order kinetics presented the highest degree of correlation coefficient (r^2^ = 0.992) among the other models. Hence, the drug release profile of *S*. *heteromalla* herbal tablets 500 mg/800 mg followed the diffusion mechanism, i.e., it ensured the constant release of the drug from the matrix into the blood throughout the delivery (Fig 3).

**Table 7:** Accelerated stability study at 40°C ± 2°C / 75% RH ± 5% RH for six months.

Parameters	Months						
	Initial	1^st^	2^nd^	3^rd^	4^th^	5^th^	6^th^
Physical appearance	Brownish green	Brownish green	Brownish green	Brownish green	Brownish green	Brownish green	Brownish green
Hardness	6.2 ± 0.05	6.2 ± 0.05	6.2 ± 0.05	6.2 ± 0.05	6.7 ± 0.05	7.0 ± 0.05	7.1 ± 0.05
Friability	0.36 ±0.14	0.36 ±0.14	0.36 ±0.14	0.36 ±0.14	0.36 ±0.14	0.36 ±0.14	0.36 ±0.14
Disintegration time	8.4± 0.11	8.4± 0.11	8.6± 0.12	8.9± 0.11	8.6± 0.12	8.8± 0.13	9 ± 0.10
Drug release	87.48	87.22	87.02	86.71	86.53	86.03	85.89

#### Accelerated stability study

The accelerated stability study of the final herbal dosage form was carried out as per ASEAN guidelines at 30°C ± 2°C/ 65 ± 5% RH and 40°C ± 2°C / 75 ± 5% RH for six months, and the parameters evaluated are mentioned in Table 7. Every month all qualitative/quantitative tests were performed, and the dosage form was found stable after the specific period.

## Discussion

This research attempted to verify scientific insight following the traditional adaptation and therefore explored the cytotoxic potential of *S*. *heteromalla* CME. Previous studies reported that anti-cancer agents of plants origin should have an IC50 value of <100 μg/ml [21]. The whole plant extract of *S*. *heteromalla* exhibited a significant inhibitory potential during the growth of HeLa cells (IC_50_ value of 62.13 μg/ml) compared to standard doxorubicin (IC_50_ value: 0.2 ± 0.03 μg/ml). Although the cytotoxic effect of *S*. *heteromalla* CME against HeLa cells is significant at 200 μg/ml, this inhibition was lower than standard doxorubicin. In our previous study, bioactivity guided fractionation and separation of *S*. *heteromalla* crude extract led to the isolation of three components arctigenin, arctiin and matairesinol. In this study, the isolated lignin (arctigenin) showed significant anti-cancer potential with IC50 of 11.04 μg/ml (doxorubicin IC50: 1 ± 0.2 μg/ml) against gynecological HeLa cancer cells while it is less toxic for normal NIH cells. Induction of cytotoxicity by *S*. *heteromalla* extract might be attributed to extraordinarily high phenolic contents, including flavonoids and arctigenin Additionally, flavonoids and arctigenin are well-known for revealing various pharmacological actions, i.e., anti-cancer and antioxidant effects. Phenolic compounds isolated from *Leptocanna chinensis* demonstrated potent antioxidant activity against cancerous HeLa cells; therefore *S*. *heteromalla* may be used as a new anti-cancer plant because of its rich source of flavonoids and arctigenin [5,22,23].

Any therapeutic/medicinal product containing single or more active constituents is designated as an herbal drug/medicine. According to WHO reports, about 80% of the world’s population depends on the drug belonging from the natural source. Many traditional herbal drug-based practices have been implemented to diagnose, prevent and cure various diseases. Several such practices have been experimentally proved that depict the scientific approach behind their traditional implementation. Minimum toxicity, improved therapeutic effect, patient compliance, and effective cost are the basis for selecting a medicine from the natural resource. The current study attempted to design *S*. *heteromalla* herbal tablets 500/800 mg. Herbal drugs, either in single or in combinations of complex matrices, contain a myriad of constituents in which no sole particular active component is responsible for the efficacy as a whole. It establishes challenging situations in quality control principles for standardizing raw materials and finished herbal dosage forms. WHO has addressed this difficulty in the planned draft (Strategic Plan for Regional Traditional Medicine of the World Health Organization) [10].

Now practically, chromatographic fingerprint analysis is used to determine the identity, consistency, and stability of herbal drugs, including selecting single or multiple components as “markers” in a chromatogram to identify and evaluate quality standards. In the present research chromatographic fingerprint analysis was employed by performing “Standard and sample (herbal tablets) solutions assay” for determining the identity, consistency and stability of *S*. *heteromalla* herbal drug. Finger print chromatograms of several peaks at variable retention times were acquired. “Marker peak” **(ShC)** at retention time 3.84 min was prominently apparent in both of the solution chromatograms (standard and sample solutions) that assured identity, consistency, and standardization of finished *S*. *heteromalla* herbal tablet dosage form. The information collected from the fingerprint was more reliable and comprehensive than the typical approach of only spotlighting the quantification of active constituents or individual markers for identity, authenticity and quantitative assay. Chromatographic fingerprint analysis was applied to determine *S*. *heteromalla* crude extract in herbal tablets by performing an assay to assure the identity and consistency of standardization of the herbal drug. The precision (Repeatability) of *S*. *heteromalla* crude extract standard solution was found as RSD: 1.15%.

About 99.15% of the drug (*S*. *heteromalla* crude extract) was recovered in herbal tablets (RSD 0.45%). The chromatograms revealed identity, and the results indicated the consistency of *S*. *heteromalla* crude extract in the finished tablet dosage form. The coefficient of variation (RSD) was statistically calculated for *S*. *heteromalla* crude extract and dosage forms, falling within the pharmacopeial acceptance criteria as acceptance limits of RSD for the dosage form and drug is not more than 3.0% and 2.0%, respectively. Powder blend had suitable flow property (Table 4) as it was free from rat holing, which was noticed by the angle of repose (32.1°) and during the granules flowing through the hopper into the die cavity during tableting. Powders with an angle of repose above 60 experienced poor flow properties. According to the USP angle of repose is employed for characterizing the optimal flow property of powder for the production of solids such as tablets and is associated with inter particulate resistance to moving.To ensure tablet weight consistency and content of the active ingredient, consistent and uniform filling of tablet dies must be accomplished Poor flow ability results in dose variations due to a variable quantity of powder flowing through the hopper into the tablet die. However, flow properties are also adjustable and controlled through different excipients like lubricants, carriers, and coating agents. Furthermore, *S*. *heteromalla* powder of crude extract flow properties was evaluated using Carr’s Index, which indirectly relates to the cohesiveness and hence to the flow property of powders. *S*. *heteromalla* crude extract Powder blend had compliance with an angle of repose. This study ensured an acceptable flow rate of *S*. *heteromalla* crude extract powder blend (10 cm^3^/s). Bulk density was found as 0.46 g/ml, which complied with the specifications as most pharmaceutical powders fall in the density range of 0.1–0.7 g/ml. Bulk density is a necessary constraint for developing methods and solid dosage form manufacturing. It determines the quantity of powder that can be fitted in a provided space of a blender, tablet press hopper, or capsule filler and capsule itself. The bulk density of the same material varies under the different dynamics courses. CI and HR were recorded as 8% and 1.21, respectively, through bulk and tapped density data. Lower CI (8%) revealed excellent flow ability and compressibility of the powder. United States Pharmacopeia (USP 42 NF 37, 2019) specifies that powders have acceptable flowing properties with Carr’s index <15% (Table 4). All herbal tablets had acceptable characteristics with little variations in friability, thickness, weight, hardness, and optimal disintegration time. Generally, tablets must have sufficient hardness to resist mechanical shock during handling and transportation. Still, it should not delay the disintegration time, which consequently influences the dissolution rate of the active loaded compounds. Different mathematical models were applied to interpret the drug release profile from the tablet matrix into the blood. The highest value of the correlation coefficient (r^2^ = 0.992) concluded the appropriate mathematical model (zero order), which followed the drug release kinetics (Rescigno, 2003).The matrix tablets of *S*. *heteromalla* herbal tablets 500 /800 mg represented a constant release profile with the polymer microcrystalline cellulose. The drug release was observed to be greatest fitted by zero order kinetics (r^2^ = 0.992), as shown in Fig 3, which implies that drug release from the matrix into the blood is constant throughout the delivery. All qualitative/quantitative tests were performed monthly to ensure drug stability through accelerated stability studies. The dosage form was found stable after a specific period.

## Conclusion

The *in vitro* results on HeLa cell line supported the ethnopharmacological relevance and traditional utilization of the *S*. *heteromalla* and established the scientific basis for the management of cervical cancer as proclaimed by traditional practitioners in China. The development of our novel chemotherapeutic herbal dosage form is intended to offer an alternate or adjuvant choice for cervical cancer patients. Furthermore, it is suggested that the potential valuable adjuvant therapies might be managed by combining herbal drugs and chemotherapeutics. Based on this *in vitro* preclinical data, further experiments can be planned to conduct pre-clinical and clinical trials using this herbal dosage form. The promising results of the trial would provide alternative treatment choices for cervical cancer patients in the future. However, *in vivo* evidence-based investigations and clinical trials are compulsory and recommended for particular clinical applications in future.
